# Stable Gene Regulatory Network Modeling From Steady-State Data ^†^

**DOI:** 10.3390/bioengineering3020012

**Published:** 2016-04-19

**Authors:** Joy Edward Larvie, Mohammad Gorji Sefidmazgi, Abdollah Homaifar, Scott H. Harrison, Ali Karimoddini, Anthony Guiseppi-Elie

**Affiliations:** 1 Department of Electrical and Computer Engineering, North Carolina A&T State University, 1601 E. Market Street, Greensboro, NC 27411, USA; jelarvie@aggies.ncat.edu (J.E.L.); mgorjise@email.arizona.edu (M.G.S.); akarimod@ncat.edu (A.K.); 2 Department of Biology, North Carolina A&T State University, 1601 E. Market Street, Greensboro, NC 27411, USA; scotth@ncat.edu; 3 Department of Biomedical Engineering, Texas A&M University, 5045 ETB, College Station, TX 77843, USA; guiseppi@tamu.edu

**Keywords:** gene regulatory network, reverse engineering, sparse network, stable network, convexity

## Abstract

Gene regulatory networks represent an abstract mapping of gene regulations in living cells. They aim to capture dependencies among molecular entities such as transcription factors, proteins and metabolites. In most applications, the regulatory network structure is unknown, and has to be reverse engineered from experimental data consisting of expression levels of the genes usually measured as messenger RNA concentrations in microarray experiments. Steady-state gene expression data are obtained from measurements of the variations in expression activity following the application of small perturbations to equilibrium states in genetic perturbation experiments. In this paper, the least absolute shrinkage and selection operator-vector autoregressive (LASSO-VAR) originally proposed for the analysis of economic time series data is adapted to include a stability constraint for the recovery of a sparse and stable regulatory network that describes data obtained from noisy perturbation experiments. The approach is applied to real experimental data obtained for the SOS pathway in *Escherichia coli* and the cell cycle pathway for yeast *Saccharomyces cerevisiae*. Significant features of this method are the ability to recover networks without inputting prior knowledge of the network topology, and the ability to be efficiently applied to large scale networks due to the convex nature of the method.

## 1. Introduction

A number of technological advances, such as oligonucleotide arrays, serial analysis of gene expression (SAGE) and cDNA microarrays [1], have enabled biomedical researchers to expeditiously and simultaneously collect large amounts of metabolomic, transcriptomic, proteomic data [2] in a single experiment, providing a wealth of information for elucidating gene regulation, functions and interactions [3,4]. Over time, repositories such as the Gene Expression Omnibus (GEO) [5] and the Biological General Repository for Interaction Datasets (BioGRID) [6] are mapping functional information and ontologies to expression data sets [2,7]. Gene expression measurement data acquired from microarray experiments typically occur in two contexts: steady-state data which provides information on interaction directions, and temporal data that allows for the investigation of temporal patterns in biological networks [8,9].

Owing to their inherent ability to encapsulate the high dimensional data of biological processes and pathways, networks have become an important tool in functional genomics [10,11]. Researchers refer to any such network that provides a system level interaction among genes as a gene regulatory network (GRN) [12,13]. GRNs are usually represented by directed graphs with nodes as genes, and edges depicting either an inhibition (negative regulation) or an activation (positive regulation) imposed by a gene over another through the production of a protein [14,15].

The process of identifying genetic interactions from measured gene expression data is referred to as reverse engineering or network inference or recovery [7]. Inferring the topology of GRNs and isolating functional subnetworks are computationally challenging tasks in contemporary functional genomics, and these efforts are valuable for advancing scientific insight and for capitalizing on the time and costs associated with experimental data [16,17,18,19]. GRNs typically contain information about the pathway to which a gene belongs and the genes it interacts with [16], and this helps to reveal potential pathway initiators and drug targets [8]. Further analysis, to map interactions among phenotypic and genotypic characteristics, can provide a framework for the identification of biomarkers for medical diagnosis and prognosis [20,21].

A plethora of modeling approaches such as co-expression clustering [22], Boolean network [23,24], Bayesian network [25] and ordinary differential equation (ODE) [8] models have been proposed for recovering genetic networks. Cluster analysis and the sequential search for patterns of gene expression related with some pathological state of interest usually provide only indirect information about the structure of the network [7]. Alternatively, grouping of co-expressed genes may be achieved using information-theoretic methods. Both approaches, however, lack causality [9]. Causality may be recovered through Bayesian networks which can handle directed graphs [9,26]. However, Bayesian networks typically do not accommodate cycles, and, hence, are unable to handle feedback motifs that are common in gene regulatory networks [26]. Causality and feedback motifs, however, are no longer a problem when the network is modeled as a set of differential equations [26]. Excellent as they are at modeling causality and feedback motifs, differential equations are only suitable for small-scale networks [9].

These existing techniques, however, rely heavily on temporal expression data which can be very difficult to acquire, and also require high computational effort [8,26]. Major considerations of sparsity, stability and causality must be captured in the biological network recovery process [2]. In this paper, the least absolute shrinkage and selection operator-vector autoregressive (LASSO-VAR) model, originally proposed for the analysis of economic time series data in [27], is adapted to include a stability constraint defined and used by [26] for the recovery of sparse and stable regulatory networks that describe steady-state data obtained from noisy perturbation experiments. The fact that LASSO-VAR is a vector autoregressive process implies that Granger causality can be inferred. The technique only requires one tuning parameter, which works to penalize non-sparse networks. The selection of this parameter is based on its mean square forecast error. The identification algorithm proposed is applicable for the identification of regulatory roles of individual genes and control genes in the network. It is also applicable for identifying genes that directly impact the bioactivity of a compound in the cell. The approach is applied to real experimental data obtained for the SOS pathway in *Escherichia coli* and the cell cycle pathway for yeast *Saccharomyces cerevisiae*. The significant features of this method are the ability to recover networks without *a priori* knowledge of the network topology, and to be efficiently applied to large scale networks due to the convex nature of the method.

## 2. Methodology

This section introduces the stable LASSO-VAR, the identification technique being adapted for reverse engineering gene regulatory networks from steady-state data [28]. In its original form, the LASSO-VAR technique described in [27] finds applications in the analysis and prediction of economic and financial time series. It is an extension of the VAR model to include a selection and shrinkage operator known as the LASSO. The inherent advantages of the LASSO-VAR are the ability to perform dimension reduction and variable selection, as well as being able to test Granger causality. For this reason, this paper adapts the LASSO-VAR concept and incorporates a stability constraint as a convex constraint to allow for the inference of a stable genetic network from steady-state data.

### 2.1. Network Identification Approach

The vector autoregressive (VAR) model is known to be one of the most flexible and easy to use models for analyzing multivariate time series [27]. It has found applications in neurosciences for the estimation of functional connectivity between several brain areas [29], and most recently in system biology for the reconstruction of gene regulatory networks [2].

In the general case, an *N*-dimensional multiple time series gene expression data $y_{1},...,y_{T}$ with $y_{t}={\left(y_{1t},...,y_{Nt}\right)}^{\prime }$ can be assumed to be generated by a stationary, stable VAR(*p*) process as [30]:

(1) $$y_{t}=v+A_{1}y_{t-1}+...+A_{p}y_{t-p}+u_{t},$$

where *p* denotes the order of the vector autoregressive process (*i.e.*, the vector autoregressive lag length), $y_{t}$ is an (N×1) random vector, $A_{i}$ is a fixed (N×N) coefficient matrix, $v={\left(v_{1},...,v_{N}\right)}^{\prime }$ is a fixed (N×1) vector of intercept terms allowing for the possibility of a nonzero mean $E(y_{t})$, $u_{t}={\left(u_{1t},...,u_{Nt}\right)}^{\prime }$ is a *N*-dimensional white noise, thus, $E(u_{t})=0$, $E\left(u_{t}u_{t}^{\prime }\right)=\Sigma_{u}$ and $E(u_{t}u_{s}^{\prime })=0$ for s≠t. The covariance matrix $\Sigma_{u}$ is assumed to be nonsingular. The framework of the general VAR(*p*) allows for the testing of Granger causality [31]. The concept of Granger causality is founded on the idea that a cause must precede an effect. This concept was originally proposed by Granger in [32].

In the present context, however, the number of genes considered in most microarray experiments generally runs from several thousands to millions, thereby making it impossible to accommodate the most general form of the Granger causality test [31]. Thus, a VAR(1) (VAR of order one) model is usually employed to allow for a pairwise comparison study as seen in [31] and [29]. Stated simply, in the case of a VAR(1), if gene *b* at time *t* is affected by a gene *a* at time (t−1), the latter should help to predict the target gene expression [29].

A first order VAR model is defined as [30]:

(2) $$y_{t}=v+Ay_{t-1}+u_{t}.$$

For convenience, (2) is usually expressed in compact matrix notation as [30]:

(3) Y=v+AZ+U,

where $Y=(y_{1},y_{2},...,y_{T})$ is an N×T data or response matrix, A is an N×N unknown coefficient matrix, $Z=(Z_{0},...,Z_{T-1})$ is an N×T covariate matrix and $U=(u_{1},...,u_{T})$ is an N×T matrix.

The solution to (3) is given as follows [30]:

(4) $$A=({\left(ZZ^{T}\right)}^{-1}Z⊗I_{N})Y,$$

where $I_{N}$ is an N×N identity matrix, and ⊗ is the Kronecker product or direct product.

In high dimensional space, the VAR processes become computationally intractable [27]. As such, the model usually contains unwanted parameters which leads to less efficient parameter estimates [33]. The LASSO-VAR model addresses the intractability issue by zeroing some elements of the coefficient matrix, which removes unnecessary variables [27,33]. The requirement that the coefficient matrix, *A*, be sparse is due to the loose connectivity that biological networks generally exhibit [8,26]. This requirement is addressed by applying an $L_{1}$ penalty to the convex least squares objective function, resulting in [27]:

(5) $$\frac{1}{2}{∥Y-v-AZ∥}_{F}^{2}+\lambda {∥A∥}_{1},$$

where ${∥X∥}_{F}^{2}=\sum_{i=1}^{m}\sum_{j=1}^{n}{\left|x_{ij}\right|}^{2}$ is the square of the Frobenius norm of **X** (*i.e.*, the sum of the absolute squares of its elements), ${∥X∥}_{1}=\Sigma_{jk}\left|X_{jk}\right|$ is the sum of the absolute values of **X**, and λ≥0 is a penalty parameter.

According to [30], if all eigenvalues of the coefficient matrix *A* of the VAR(1) process have absolute values less than 1, the sequence $A^{i},i=0,1,...$ is absolutely summable; as such, the infinite sum $\sum_{i=1}^{\infty }$ exists in mean square. Hence, in general, a VAR(1) is said to be stable iff all eigenvalues of *A* have absolute value less than one. It is mathematically equivalent to [30]:

(6) $$det(I_{N}-A\tau )\neq 0\mathrm{for}|\tau |\leq 1.$$

The original formulation of the LASSO-VAR technique by [27] lacks the ability to infer a stable network. This setback means that stability cannot be inferred from gene perturbation experiments. To solve this inadequacy, a stability constraint that relies on the theorem by Geršgorin as discussed in [34] is incorporated into the LASSO-VAR objective function (5).

Geršgorin’s theorem states that all the eigenvalues of an n×n matrix $A=(a_{ij})$ are in the union of the discs whose boundaries are circles $C_{1},C_{2},...,C_{n}$ with centers at the points $a_{11},a_{22},...,a_{nn}$ and the radii are: $r_{i}=\sum_{j=1,j\neq i}^{n}\left|a_{ij}\right|$. Stated more compactly, every eigenvalue *τ* must be contained in at least one of the circles characterized by the rows of *A* for an n×n matrix *A*. In essence, the eigenvalues of a square matrix can not be too far from its diagonal entries. It follows that [34]:

(7) $$|\tau -a_{ii}|\leq \sum_{j=1,j\neq i}^{n}\left|a_{ij}\right|,\;i=1,2,...,n.$$

As such, the real part of each eigenvalue must satisfy one of the conditions [34]

(8) $$\text{Re}\left[\tau \right]\leq a_{ii}+\sum_{j=1,j\neq i}^{n}\left|a_{ij}\right|,\;i=1,2,...,n.$$

Since $V^{-1}AV$ and *A* have the same eigenvalues for all invertible matrix *V*, it is possible to apply Geršgorin’s theorem to $V^{-1}AV$. For a good choice of *V*, one can find some tighter bounds for the eigenvalues. [26]. A particularly convenient choice is $V≜\text{diag}(v_{1},...,v_{n})$, with $v_{i}>0$ for all i=1,...,n. Then, $V^{-1}AV=\left(v_{j}a_{ij}/v_{i}\right)$. It follows therefore that, ∀V∈V, the real part of an eigenvalue of *A* must satisfy [26]:

(9) $$\text{Re}\left[\tau \right]\leq a_{ii}+\sum_{j=1,j\neq i}^{n}\frac{v_{j}}{v_{i}}\left|a_{ij}\right|,\;i=1,2,...,n.$$

The stability requirement of the algorithm stems from the steady-state nature of the gene expression data adopted. Stability of the network simply refers to the robustness of the network to topology and parameter changes, as well as instrumental and biological noise [35]. The inherent stability description of the VAR model therefore allows the incorporation of a stability constraint that helps to address the specification of a stable gene regulatory network from steady-state data.

Incorporating this concept as a constraint retains the convex nature of the objective function; hence, it has the associated properties of scalability and global optimality. The resulting overall optimization problem is given as:

(10) $$\begin{array}{cc} minimize & \frac{1}{2}{∥Y-AZ∥}_{F}^{2}\\ subject\;to & {∥A∥}_{1}<\lambda ,\;0\leq \lambda \leq 1\\ & a_{ii}\leq -\sum_{j=1,j\neq i}^{n}\frac{v_{j}}{v_{i}}\left|a_{ij}\right|,\;i=1,...,n,\;v_{i},\;v_{j}>0 \end{array}.$$

The choice of $v_{i}$ is dependent on the stability requirements defined in the problem formulation. Zavlanos *et al.* [26] provide a convenient way to choose the weights $v_{i}$.

**Figure 1:**
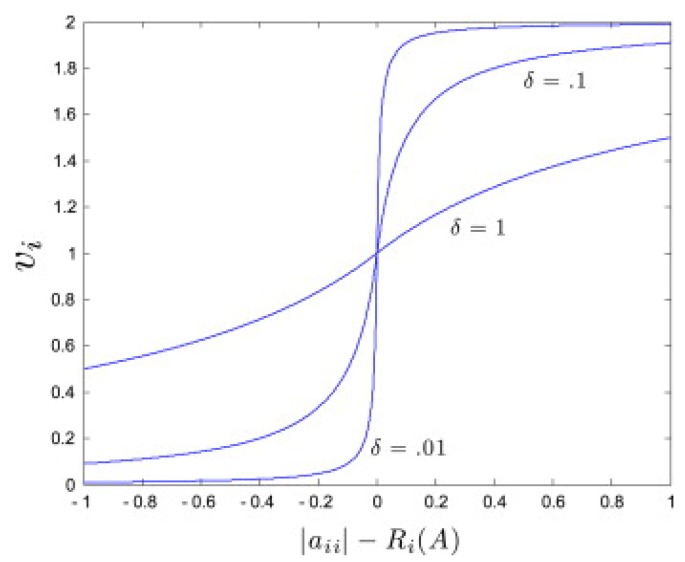
Plot of $v_{i}$ as a function of the entries $|a_{ii}|-R_{i}\left(A\right)$, for average β=0 and different values of the parameter 0<δ≤1. Taken from [26].

Define the deleted absolute sum for row *i* as $R_{i}\left(A\right)≜\sum_{j\neq i}\left|a_{ij}\right|$. Then, for $\beta ≜\frac{1}{n}\sum_{i=1}^{n}\left(\right|a_{ii}\left|-R_{i}\left(A\right)\right)$ the weights $v_{i}$ are chosen given Figure 1 as follows [26]:

(11) $$v_{i}≜\left\{\begin{array}{cc} 1+\frac{|a_{ii}|-R_{i}\left(A\right)-\beta }{\delta +(|a_{ii}|-R_{i}\left(A\right)-\beta )}, & \text{if}\;|a_{ii}|-R_{i}\left(A\right)>\beta \\ \frac{\delta }{\delta -(|a_{ii}|-R_{i}\left(A\right)-\beta )}, & \text{if}\;|a_{ii}|-R_{i}\left(A\right)\leq \beta \end{array}\right..$$

Solving the constrained optimization problem in (10) iteratively yields a sparse, stable coefficient matrix that models the causal interactions among the genes under observation as desired.

In shrinkage problems, a formalized approach for the selection of an optimal penalty parameter value is achieved by employing either a *k*-fold or a leave-one-out cross validation [27]. Due to time-dependence, however, traditional cross-validation techniques are not well-suited for the problem formulation. The optimal penalty parameter is selected by minimizing the one-step ahead mean square forecast error (MSFE) [27]. This process starts by dividing the data into three periods: one for initialization, one for training, and one for forecast evaluation. Two time indices are defined as: $T_{1}=\left\lfloor \frac{T}{3}\right\rfloor$, and $T_{2}=\left\lfloor \frac{2T}{3}\right\rfloor$, where ⌊x⌋ is the largest integer less than or equal to *x* . The period $T_{1}+1$ through $T_{2}$ is used for training and $T_{2}+1$ through *T* for the evaluation of forecast accuracy in a rolling manner. In addition, for the one-step ahead forecast based on all observations from 1,...,t is defined as ${\hat{y}}_{t+1}^{\lambda }$ [27]. The objective therefore is to minimize [27]:

(12) $$MSFE\left(\lambda \right)=\frac{1}{T_{2}-T_{1}-1}\sum_{t=T_{1}}^{T_{2}-1}{∥{\hat{y}}_{t+1}^{\lambda }-y_{t+1}∥}_{F}^{2}.$$

MSFE represents the most appropriate criterion given the use of the least squares objective function. Instead of parallelizing the cross-validation procedure, this approach uses the result from the previous period as an initialization, substantially reducing computational time [27].

The algorithm was implemented in MATLAB^®^ R2014a using the cvx 2.1 toolbox for convex optimization problems [36].

### 2.2. Performance Evaluation

In order to evaluate the performance of the proposed network identification algorithm for reconstructing stable gene regulatory networks from datasets, statistical measures are employed. For predictive analysis, the confusion matrix (Table 1), represents a table with two rows and two columns that report the number of True Positives (TPs), False Positives (FPs), True Negatives (TNs) and False Negatives (FNs). Since the parameter, *λ*, regulates the weight imposed on sparsity, the terms “Positives” and “Negatives” here refer to non-zero and zero interactions between genes, respectively.

TP represents the interaction that exists in both the true network and inferred network, FP denotes the interaction that does not exist in the true network but was falsely inferred, TN is the interaction that does not exist in either the true network or the inferred network, while FN represents the interaction that does exist in the actual network but is not recovered by the network identification method.

Three other criteria *Sensitivity* (sen), *Specificity* (spc) and *Precision* (not commonly used) are also employed as evaluation methods. *Sensitivity* (sen), is the fraction of the number of recovered true regulations to all regulations in the model. *Specificity* (spc), is the ratio of correctly found no-interactions to all no-interactions in the model. *Precision* (pre), measures the fraction of the number of correctly found regulations to all found regulations in the inferred network. These three performance criteria are defined as follows [37]:

(13a) $$\begin{array}{c} \text{Sensitivity}=\frac{TP}{TP+FN}, \end{array}$$

(13b) $$\begin{array}{c} \text{Specificity}=\frac{TN}{TN+FP}, \end{array}$$

(13c) $$\begin{array}{c} \text{Precision}=\frac{TP}{TP+FP}. \end{array}$$

For the purposes of comparison with other network identification techniques, the performance evaluation graphs are restricted to sensitivity and specificity.

Overall, the following specific steps are performed for modeling the GRN using LASSO-VAR. The time series of gene expressions are converted to a matrix where the rows are expression of various genes and the columns are observations at different time points. Assuming the model of Equation (2), the optimization problem as Equation (10) can be generated. Solving this optimization using the iterative approach yields the matrix A with sparse and stable structure. The optimal values of hyperparameters ( *λ* and $v_{i}$ ) should also be found. The structure of gene regulatory network is found using the matrix A. The results of our method are then compared with target GRNs that are accepted or inferred by other means.

Data for GRN recovery of the *Escherichia coli* SOS network was from a perturbation experiment for relative RNA expression changes from Table S6 of [8]. Data for GRN recovery of the cell cycle pathway in yeast *Saccharomyces cerevisiae* was based on the alpha time series of [38], including 18 time points at 7 min interval over 119 min. Data came from the yeast cell cycle analysis database [39] with the analysis conducted on a set of 14 genes. Standard (and systematic) gene names for these genes are: FUS3 (YBL016W), SIC1 (YLR079W), FAR1 (YJL157C), CDC6 (YJL194W), CDC20 (YGL116W), CDC28 (YBR160W), CLN1 (YMR199W), CLN2 (YPL256C), CLN3 (YAL040C), CLB5 (YPR120C), CLB6 (YGR109C), SWI4 (YER111C), SWI6 (YLR182W) and MBP1 (YDL056W).

## 3. Results and Discussion

In this section, the efficiency of the proposed network identification is analyzed by studying networks for which the experimental data as well as the ground truth is available. The studied datasets consist of real experimental dataset in a known subnetwork of the SOS pathway in *Escherichia coli*, provided in [26], and the cell cycle pathway in yeast *Saccharomyces cerevisiae*.

With datasets that have a known network, it is possible to evaluate the performance of the algorithm, allowing for the measurement of the false positives, false negatives, *etc*. The effect of different values of the penalty parameter *λ* on the performance of the algorithm is also investigated.

### 3.1. SOS Pathway in Escherichia coli

The proposed identification algorithms is first applied to a sub-network of the SOS pathway in *Escherichia coli*, using the gene perturbation experimental data set provided in [8]. The SOS pathway is known to control the survival and repair of cells after DNA damage [8]. This pathway typically involves the genes *recA* and *lexA* directly regulating over 30 genes, and indirectly controlling over 100 genes [8].

**Figure 2:**
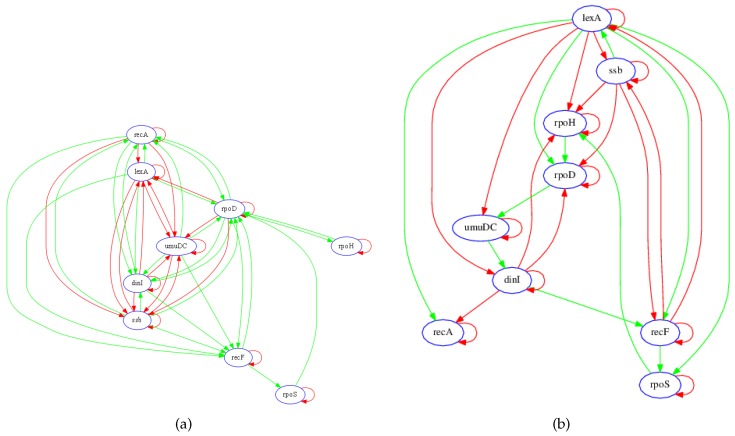
Known and recovered GRN for SOS pathway in *E. coli*. (**a**) Diagram of interactions in the SOS network. DNA lesions caused by mitomycin C (MMC) (**blue hexagon**) are converted to single-stranded DNA during chromosomal replication. Upon binding to ssDNA, the RecA protein is activated (RecA*) and serves as a coprotease for the LexA protein. The LexA protein is cleaved, thereby diminishing the repression of genes that mediate multiple protective responses. Green arrows denote positive regulation, while red arrows denote negative regulation. *Adapted from* [8]. (**b**) Diagram of the recovered gene regulatory network of the SOS pathway in *Escherichia coli*. Green arrows denote positive regulation, while red arrows denote negative regulation.

The sub-network considered, shown in Figure 2a, consists of nine genes and several transcription factors and metabolites [8] whose expression levels are measured over nine different perturbations. According to Gardner *et al.* [8], the nine transcripts in the test network (Figure 2a) were chosen to enable evaluation of the performance of their proposed algorithm. These nine transcripts include the key mediators of the SOS response (*lexA* and *recA*) and sets of genes with known regulatory roles (*ssb*, *recF*, *dinI*, and *umuDC*) and unknown regulatory roles (*rpoD*, *rpoH*, and *rpoS*). The presence of genes with regulatory roles that are already known allows this network to be used to validate an inference algorithm [8].

#### 3.1.1. Network Recovery

In order to evaluate the performance of the proposed algorithm, those links that are correctly recovered in the model are determined based on knowledge of the true network. An inferred connection is regarded to be accurate if there exists a known RNA, protein, or metabolite pathway between the two transcripts, and if the sign of the net effect of regulatory interaction (*i.e.*, inhibition or activation) is correct, as determined by the known network in Figure 2a. In general, since RNA concentrations (*i.e.*, expressions) were measured and not metabolite or protein species, the recovered regulatory network model does not necessarily depict physical connections; rather, the links show effective functional associations between transcripts.

Figure 2b shows the inferred gene regulatory network from the steady-state data. The identification algorithm accurately identified the key regulatory associations in the network. For instance, the model correctly shows that *lexA* activates *recA* while negatively regulating its own transcription, whereas *recA* negatively regulates its own transcription. In addition, the model identified *lexA* as having the greatest regulatory influence on the other genes in the network. Due to the differences in network topology (e.g., *recA*, *lexA* and *CDC20*), inaccuracies are expected from either the current published model, the LASSO-VAR GRN recovery, or both. Some of these potential differences may alternatively be dependent on the dynamic state of the system as inferred from the temporal context.

**Figure 3:**
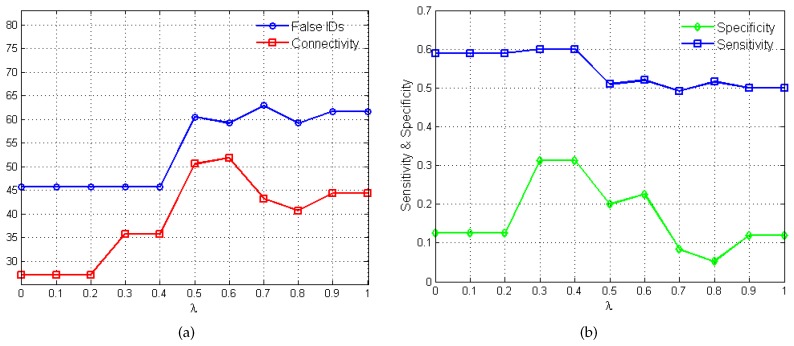
Variations in *λ* and the corresponding algorithm performance. (**a**) plot of *λ* *versus* total number of false identifications and net connectivity in percentages. (**b**) plot of *λ* *versus* sensitivity and specificity.

The plots in Figure 3 show the variations in algorithm performance as the penalty parameter *λ* varies between 0 and 1. In Figure 3a, the total number of false identifications (*i.e.*, false activations, inhibitions and no-interactions) and the net connectivity of the network are measured against the different *λ* values. The net connectivity provides a measure of the total number of interactions inferred by the algorithm. As such since sparsity is required, lower values in the net connectivity is desired. Figure 3b shows the variations in the performance metrics, sensitivity and specificity, as *λ* changes.

The choice of the "best" penalty parameter for the given application represents the *λ* value that produces the best trade-off between the number of false identification, net connectivity, sensitivity and specificity. In this regard, values of 0.3 and 0.4 produce 46% false identification, 37% net connectivity, sensitivity of 60% and specificity of 31%. Eventually, λ=0.3 is selected based on its superior MSFE value of 5.20 as opposed to 5.22 for λ=0.4 and satisfies the desired constraints of stability, sparsity and causality.

Table 2 shows how the proposed network identification algorithm without *a priori* knowledge of the network structure compares with that proposed by Zavlanos *et al.* [26] with 30% *a priori* knowledge of the network.

Table 2 shows how the proposed algorithm compares to that proposed by Zavlanos *et al.* In all, the recovered network has four false activations, eight false inhibitions, 25 false no-interactions, and 37 false identifications in total, while satisfying the desired constraints of stability, sparsity and causality. The penalty parameter, *λ* selected is 0.4 based to its MSFE value.

### 3.2. Yeast Saccharomyces Cerevisiae Cell Cycle

**Figure 4:**
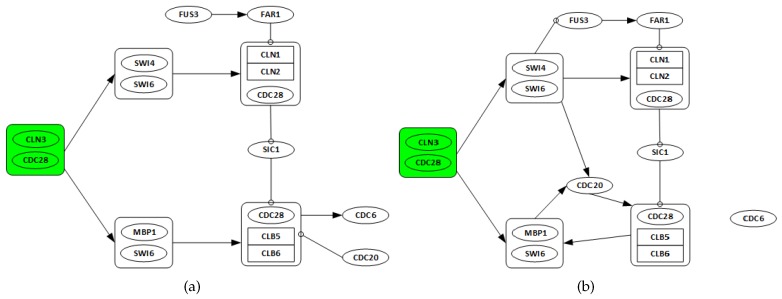
Known and recovered GRN for cell cycle pathway in yeast *Saccharomyces cerevisiae*. (**a**) target pathways of the 14 genes. CDC28 associates with cyclin CLN3 at the start of mitosis to cause the activation of SBF (SWI4/SWI6) and MBF (MBP1/SWI6), promoting the transcription of CLN1, CLN2. At G1 phase CDC28 associates with G1-cyclins CLN1 to CLN3, while B-type cyclins CLB1 to CLB6 regulate CDC28 during S, G2, and M phases. CLN1 and CLN2 interacting with CDC28 promote activation of B-type cyclin associated Cyclin-dependent kinase (CDK), which drives DNA replication and entry into mitosis. *Adapted from* [41]. (**b**) recovered yeast cell cycle pathway. The arrows show the direction of regulation. Some key regulations like activation (positive regulation) of the SBF (SWI4/SWI6) and MBF (MBP1/SWI6) complexes by the starter complex (CDC28/CLN3) are recovered.

From 1998, when Spellman *et al.* published the yeast *Saccharomyces cerevisiae* (*i.e.*, budding yeast) cell cycle microarray expression levels [38], many computational methods have been applied to these data. To demonstrate the applicability of the proposed algorithm in this study, a subset from yeast *Saccharomyces cerevisiae* microarray time series dataset including 14 genes, FUS3, SIC1, FAR1, CDC6, CDC20, CDC28, CLN1, CLN2, CLN3, CLB5, CLB6, SWI4, SWI6 and MBP1, is perturbed and used. The details of *S. cerevisiae* cell cycle control are well known, as shown in Figure 4a.

The 14 genes are known to be involved in the early cell cycle of the yeast *Saccharomyces cerevisiae*. The cell cycle describes the series of events that precedes its division and duplication [40]. The mitotic cell cycle in yeast is accomplished through a reproducible sequence of events: DNA replication (S phase) and mitosis (M phase) separated temporally by gaps, G1 and G2 phases. At the G1 phase, CDC28 associates with CLN1, CLN2 and CLN3, while CLB5 and CLB6 controls CDC during S, G2, and M phases [41]. Cell cycle progression begins upon the activity of CLN3/CDC28. When the levels of CLN3/CDC28 accumulate more than a certain threshold, SWI4/SWI6 and MBF1/SWI6 are activated, promoting transcription of CLN1 and CLN2 [41]. CLN1/CDC28 and CLN2/CDC28 promote activation of other associated kinase, which drives DNA replication. SIC1 and FAR1 are the substrates and inhibitors of CDC28. CDC6 and CDC20 affect the cell division control proteins. Mitogen-activated protein kinase affect this progression through FUS3 [41]. The dataset generated by Spellman *et al.* [38] contains three time series measured using different cell synchronization methods: *α* factor-based arrest (referred to as alpha, includes 18 time points at 7 min interval over 119 min), size-based (*elu*, 14 time points at 30 min interval over 390 min), and arrest of a cdc15 temperature-sensitive mutant (*cdc15*, 24 time points, the first four and last three of which are at 20 min interval and the rest are at 10 min interval over 290 min). The *alpha* dataset is used and then studied in more detail as has been explored in literature [42].

Data for the expected topology of this network were extracted from the Kyoto Encyclopedia of Genes and Genomes (KEGG) [41], which is a major collection of knowledge for molecular and genetic pathways and includes information on experimental observations in organisms. The KEGG regulatory pathway represents current knowledge on the protein and gene interaction networks.

#### 3.2.1. Network Recovery

As discussed in the preceding section, the KEGG pathway is considered as the target network for comparison. Complexes including one or several genes are considered as a ‘*gene*’ in the network. There are 10 complexes, including CLN3/CDC28, SWI4/SWI6, MBP1/SWI6, CLN1/CLN2/CDC18, and CLB5/CLB6/CDC28. Other nodes that are made of one single gene only, CDC20, CDC6, SIC1, FAR1, and FUS. The following assumptions are made:

1. Genes CLN3 and CDC28 are only considered as possible regulators, as they are starters of the cell cycle network.
2. All discovered links from any gene in one complex to any other genes in a different complex are considered as a single regulation.
3. All regulations among genes in the same complex are ignored.

Figure 4b is the recovered network from the perturbed *alpha* gene expression data for the yeast *Saccharomyces cerevisiae* cell cycle pathway.

**Figure 5:**
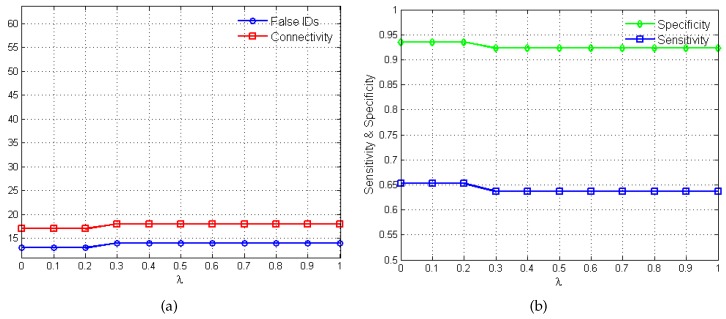
Variations in *λ* and the corresponding algorithm performance. (**a**) plot of *λ* *versus* total number of false identifications and net connectivity in percentages; (**b**) plot of *λ* *versus* sensitivity and specificity.

The recovered network has seven true positives and five false positives. The algorithm recovers key regulations. For instance, the activation (positive regulation) of the SWI4/SWI6 and MBP1/SWI6 complexes by the starter complex are identified. The graphs in Figure 5 show the changes in algorithm performance for varying *λ* values. In Figure 5a, the relationship between *λ* and total false identification as well as the net connectivity of the network. The plot in Figure 5b show the variations in *λ* and the corresponding effects on the performance of the algorithm. Again, the terms false identifications and net connectivity have the same meanings as discussed in Section 3.1.1. The results in this application are quite uniform due to the assumptions made in grouping some genes into complexes as required. λ∈[0,0.2] provides the best trade-off between the number of false identification, net connectivity, sensitivity and specificity. They produce sensitivity and specificity values of 65% and 94%, respectively. Based on its lower MSFE value, λ=0.2 is chosen.

## 4. Conclusion and Discussion

In this paper, the least absolute shrinkage and selection operator—vector autoregressive (LASSO-VAR) model–has been adapted in solving the problem of identifying a minimal model that best explains genetic perturbation data obtained at a network’s equilibrium state. The fact that the network identification algorithm is an autoregressive technique means it has the inherent ability to model Granger causality, making it possible to identify regulatory roles among the genes under consideration. Additionally, the technique handles the sparsity constraint imposed by the loose-connectivity restriction of biological networks through the application of the $L_{1}$ penalty term. Due to the steady-state nature of the expression data, a stability constraint is imposed on the original LASSO-VAR objective function, which allows robustness of the inferred networks to slight variations in the input.

To evaluate the reliability and efficiency of LASSO-VAR for recovering stable, sparse and causal regulatory interactions from steady-state gene expression data, data from the SOS pathway in *E. coli* was first used. The performance of the algorithm was measured and compared with results obtained in literature. This comparison was based on two statistical evaluation criteria, sensitivity and specificity, which allowed the accuracies of the inferred network structure using the LASSO-VAR technique to be quantified. LASSO-VAR performed without prior knowledge at a roughly comparable level to the alternative Zavanos method that requires some prior knowledge. The efficiency of the stable LASSO-VAR for learning the network structure was then evaluated using the perturbed gene expression data of 14 genes in yeast *Saccharomyces cerevisiae* cell cycle reported in [41]. The network inferred from the yeast data by LASSO-VAR is compared with the known network from the cell cycle pathway of the yeast *Saccharomyces cerevisiae* using the evaluation criteria: sensitivity and specificity. Results showed the ability of the identification algorithm to infer the regulatory network for the cell cycle pathway.

The surge of large biological data sets [43,44] and the aggressive efforts at methods for ontology-driven annotation and data modeling [45,46] have helped to provide opportunities for a more objective and reproducible basis for analysis. The formulation of our pathway recovery analysis model fulfills those criteria necessary for it to be highly scalable with data for biological networks by: (1) avoiding the context-limiting aspects of *a priori* knowledge and presumptions of frequentist-type statistics which are difficult to implement based on the inherent sparsity of biological networks; and (2) being strictly data-driven in matrix-based numerical forms with one controlling parameter—*i.e.*, being outside of subjective standards of knowledge and curation. The criterion for stability as we investigate it as a necessary condition for steady-state data, although seemingly trivial, is nonetheless fundamental for how structural and functional robust aspects for pathways are identified. In general, scoring outcomes from our methodology identified key mechanisms of pathways through a scoring gradient. We expect there to be significant dividends from this approach that go beyond our initial usage of a reference database for tallying of true *versus* false results. The quantitative support underlying false positive and false negative results for the target model may aid in the development and testing of new hypotheses, or quality control measures, agendas that are important in large-scale investments into empirical data collection such as for perturbation studies.

Canonical pathways provide well-vetted models that have a deep legacy in empirical published studies [46,47]. It is still the case, however, that uncharted dynamics and natural variation go beyond the limited range of studied organisms and would furthermore impact predictive modeling even for the two chosen models of this study for which knowledge remains limited [48,49]. For instance, synchronization of expression across multiple genes is likely lost following its initial measurement at a starting point of an experimental assay. The gain or loss of known interactions as input data would furthermore be expected to impact the predictive power of LASSO-VAR. There is, therefore, a need to more systematically study LASSO-VAR across these frontier contexts involving a larger number of simulated and empirical data sets. This would in particular aid the empirical study of computational performance, beyond theoretical expectations for restricting the parameter space through the penalty parameter [27]. This would also provide a robust capacity for selecting which genes to base a model upon and for constructing the analysis in a way that separates initialization and training from forecast evaluation, to guide contrasting modes of usage for how LASSO-VAR could be used in supervised *versus* unsupervised modes of analytical evaluations. As multiple gene expression data resources and annotations may be harnessed for this effort [50,51,52,53], it remains an essential next step to identify computational and theoretical limits for objectively inferring GRN configurations based on input data complexity and sizes. The overall outcome for such an effort would help resolve the lack of overlap between pathway databases and approaches to analytical treatment, both with respect to content [50,54] and foundational criteria such as how to define start and stop points for individual pathways [50]. Future usage of this approach could identify pathways for assembly and disassembly of differentially constructed multicomponent cellular objects recovered at the same point of time. Such usage of cellular component data to infer subunit associations would add to the explanatory potential of this algorithm, and help to guard against the *post hoc, ergo propter hoc* fallacy for how changes in cellular composition would otherwise be inferred from analyses conducted only upon time series.

## Figures and Tables

**Table 1:** Confusion matrix.

	True Network		Total
Inferred Network	True Positive	False Postive	**P’**
	False Negative	True Negative	**N’**
Total	**P**	**N**	

**Table 2:** Comparisons of the inferred network for the SOS pathway in *E. coli* using LASSO-VAR and Zavlanos’ method.

	TP	FP	TN	FN	Sensitivity	Specificity	Precision
LASSO-VAR	39	11	5	26	60%	31%	78%
ZAVLANOS [26]	40	10	15	16	71%	60%	80%
